# The role of the hypothalamic Lhx6 GABAergic neurons in REM sleep control

**DOI:** 10.1093/sleep/zsad331

**Published:** 2023-12-30

**Authors:** Amarine Chancel, Patrice Fort, Pierre-Hervé Luppi

**Affiliations:** Université Claude Bernard Lyon 1, CNRS, INSERM, Centre de Recherche en Neurosciences de Lyon CRNL U1028 UMR5292, SLEEP Team, Bron, France; Université Claude Bernard Lyon 1, CNRS, INSERM, Centre de Recherche en Neurosciences de Lyon CRNL U1028 UMR5292, SLEEP Team, Bron, France; Université Claude Bernard Lyon 1, CNRS, INSERM, Centre de Recherche en Neurosciences de Lyon CRNL U1028 UMR5292, SLEEP Team, Bron, France

A large body of data showed that although the brainstem is necessary and sufficient to generate REM sleep, the lateral hypothalamic area (LHA) and the ventral zona incerta (vZI) contain GABAergic and MCH neurons controlling the state by descending projections [1, 2]. Indeed, a very large number of cFos-positive cells was observed in these regions after REM sleep rebound in rats [3]. Around 75% of these cFos-labeled cells express GAD67 mRNA and are likely GABAergic [2]. Interestingly, it was then shown that 45% of the GABAergic neurons located in the mouse vZI express the LIM homeodomain factor (Lhx6) and are distinct from the REM-active MCH and the wake–active hypocretin-orexin neurons [4]. It has also been shown that Lhx6 vZI neurons increased cFos expression at lights on compared to lights off [4]. We then more specifically showed that around 10% of the Lhx6 neurons in vZI are activated during REM sleep rebound occurring after deprivation in mice and that these neurons constitute one-third of the cFos + neurons localized in the vZI [5]. Only 2% of the Lhx6 neurons in vZI were activated during wake. Altogether, these results suggested that Lxh6 neurons in vZI could be involved in REM sleep but a direct demonstration was needed. In their paper published in this issue, Vidal-Ortiz et al. [6] filled this gap by confirming using calcium imaging that most Lhx6 neurons are active during REM sleep and by demonstrating using optogenetics that they indeed play a role in REM sleep control. In the first part of their study, Vidal-Ortiz et al. [6] recorded specifically the activity of Lhx6 neurons in vZI using calcium imaging in Lhx6-cre mice. They found that 45.5% of the neurons recorded were maximally active during REM sleep and started firing 15 seconds before its onset. Around 30% were maximally active during wake, 11% during wake and REM sleep, and 9% during NREM sleep. Interestingly, the NREM neurons were also activated 15 seconds before sleep onset. These results fit with the previous finding that a large proportion of GABAergic (vesicular GABAergic transporter, vGAT positive) neurons in ZI and LHA discharge maximally during REM sleep [7, 8]. Furthermore, in agreement with the present data, these neurons anticipate REM sleep onset indicating that they could play a role in triggering the state.

In the second part of Vidal-Ortiz et al. [6] study, Lhx6 neurons were optogenetically stimulated unilaterally 1 minute on and 4 minutes off for 4 hours at 1 HZ or 5 Hz. It induced a significant increase in both REM sleep quantities and the number of REM bouts. These results indicate that despite the fact that the population of Lh6x neurons in vZI is functionally heterogeneous, their global optogenetic activation significantly facilitates the expression of REM sleep, without any impact on wake and NREM.

In partial agreement with this study, it has been previously shown that mice with a conditional deletion of Lhx6 in the hypothalamus show an increase in wake time and a reduction of NREM and REM sleep relative to controls [4]. The decrease in REM sleep was greater than that of NREM sleep. In the same study, it was further shown that chemogenetic activation of Lhx6 + neurons strongly increased REM sleep and slightly increased NREM and decreased wake during 12 hours following CNO injection. Importantly, the effect on NREM and wake but not that on REM sleep was abolished after injection of the hypocretin receptor antagonist suvorexant indicating that the hypocretin-orexin neurons are involved [4]. In contrast, chemogenetic inhibition of Lhx6 neurons during the light phase induced a decrease in NREM and REM sleep and an increase in wake [4]. The optogenetic data of Vidal-Ortiz et al. [6] only partly fit with these experiments since they reported only an effect on REM sleep.

In summary, the present paper combined with previous data convincingly shows that Lhx6 GABAergic neurons are implicated in REM sleep control. The open question is how they influence REM sleep and what is their specific role compared with the MCH neurons previously discovered.

It has indeed been previously shown with unit recordings in head-restrained rats that MCH neurons fire specifically during REM sleep [9]. These results were later confirmed by calcium imaging data showing that these neurons are indeed less active during NREM sleep and quiet wake compared to REM sleep [10–12]. It has been further shown that MCH neurons localized in the LHA and the vZI are cFos-positive after REM sleep rebound but not wakefulness [3]. The increase in sleep reported for the Lhx6 neurons was also obtained using chronic (24 hours) optogenetic activation of MCH neurons [13]. In addition, their acute optogenetic activation at the onset of NREM did not increase NREM duration but increased the probability of NREM-to-REM transitions. This effect was also obtained using chemogenetics to modulate the activity of MCH neurons [14]. When optogenetic stimulations were applied at REM sleep onset, the duration of REM sleep episodes was significantly prolonged [15]. However, in contrast to the Lhx6 neurons that express vGAT, it has recently been shown that MCH neurons express the vesicular glutamate transporter 2 (vGlutT2) [16, 17] strongly suggesting that they are glutamatergic. Altogether, these results indicate the existence of two different populations of REM sleep-inducing neurons in the vZI and LHA. The question still open is what are the respective roles and the downstream targets of these two populations?

**Figure 1.**
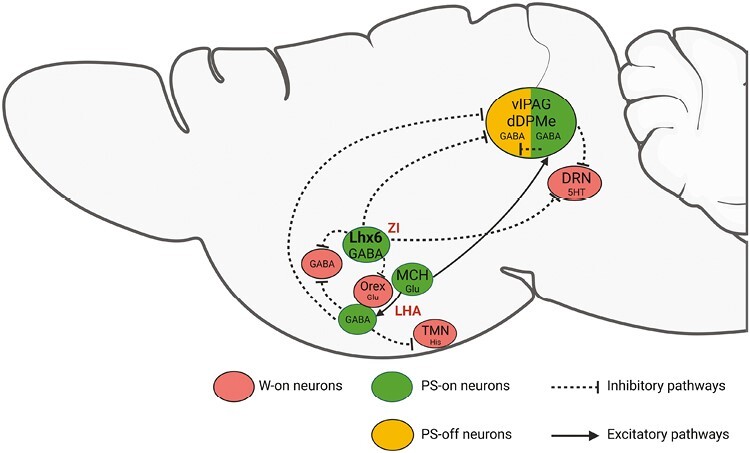
Two populations of neurons localized in the ventral zona incerta (vZI) and the lateral hypothalamic area (LHA), the Lhx6 and MCH neurons are active during REM sleep. An additional population of GABAergic neurons is activated during REM sleep. We propose that during REM sleep, Lhx6 neurons inhibit Wake–active hypocretin and GABAergic neurons of the HLA as well as the REM-off dorsal raphe Wake neurons and the GABAergic neurons localized in the ventrolateral periaqueductal gray (vlPAG) and the dorsal deep mesencephalic reticular nucleus (dDPMe). In contrast, the MCH-glutamatergic neurons would excite GABAergic REM-on neurons localized in the LHA and the vlPAG and by this mean inhibit the wake histaminergic neurons of the tuberomammillary nucleus (TMN) and the REM-off neurons of the vlPAG/dDPMe.

It has been shown that the Lhx6 neurons project to lateral hypothalamic hypocretin and GABAergic neurons and dorsal raphe serotonergic (DRN) wake–active neurons and to the ventrolateral periaqueductal gray (vlPAG) [4] known to contain the GABAergic neurons gating REM sleep [18]. These projections could therefore play a role in the induction of REM sleep by Lhx6 neurons [4](Figure 1). Furthermore, the GABAergic inhibitory nature of the projections of the Lhx6 neurons to hypocretin and neighboring GABAergic neurons within the LHA has been directly confirmed using photostimulation of Lhx6 neurons in brain slices [4]. Interestingly, Lhx6 neurons also receive inputs from wake–active and REM sleep-inhibiting neurons such as the DRN serotonergic neurons and the GABAergic neurons of the vlPAG. They also receive inputs from cholinergic neurons of the basal forebrain and pedunculopontine nucleus [4]. These projections could inhibit Lhx6 neurons during wake.

The MCH neurons might also promote REM sleep by means of their descending pathway to the REM-off vlPAG GABAergic neurons and the histaminergic wake–active neurons. Indeed, 40% of the neurons projecting from the LHA and vZI to the vlPAG express MCH [1] and inhibition of MCH terminals in the vlPAG significantly reduced transitions into REM sleep [19]. In addition, optogenetic activation of MCH axons in the tuberomammillary nucleus (TMN) inhibits histaminergic cells through activation of GABA_A_ receptors [15]. Since MCH neurons are now believed to be excitatory glutamatergic neurons, they might excite local inhibitory REM-on GABAergic neurons themselves inhibiting the above REM-inhibiting systems (Figure 1).

In summary, the present work of Vidal-Ortiz et al. [6] convincingly demonstrates that around half of the Lhx6 neurons are involved in REM sleep control. These results combined with previous ones clearly show that at least two different populations of vZI and LHA neurons, the Lhx6 and the MCH neurons, promote REM sleep. The fact that Lhx6 but not MCH neurons anticipate REM sleep onset might indicate that the former but not the latter are involved in REM sleep induction. Additional studies are strongly required to confirm that the MCH and Lhx6 are respectively glutamatergic and GABAergic neurons acting therefore on different downstream systems to promote REM sleep as proposed in Figure 1. The upstream systems controlling the activation of these neurons during REM sleep remain also to be identified. Furthermore, there is still room for additional neuronal populations controlling REM sleep within vZI and LHA since these two populations constitute only a portion of all cFos-labeled neurons in these regions after REM sleep rebound [3]. Finally, the results of Vidal-Ortiz et al. [6] also show that Lhx6 is not homogeneous with a minority of them being active during NREM or wake. Such heterogeneity is difficult to interpret in terms of function and requires additional exploration.
